# Experimental Study on Thermal Conductivity of Water-Based Magnetic Fluid Loaded with Different Nanoparticles

**DOI:** 10.3390/nano13222952

**Published:** 2023-11-15

**Authors:** Zhe Su, Yanhong Cheng, Zhifeng Liu, Jiayi Zhou, Decai Li, Ying Li

**Affiliations:** 1 Institute of Advanced Manufacturing and Intelligent Technology, Beijing University of Technology, Beijing 100124, China; suzhe@emails.bjut.edu.cn (Z.S.); zhoujy@emails.bjut.edu.cn (J.Z.); li_ying@bjut.edu.cn (Y.L.); 2 Key Laboratory of Advanced Manufacturing and Intelligent Technology for High-End CNC Equipment, Jilin 130015, China; 3 State Key Laboratory of Tribology, Tsinghua University, Beijing 100084, China; lidecai@tsinghua.mail.edu.cn

**Keywords:** magnetic fluid, thermal conductivity, experimental measurement, carbon nanotubes, theoretical model

## Abstract

Magnetic fluids, a new type of energy transfer fluid with tunable properties, have garnered significant interest from researchers worldwide. Hybrid magnetic fluids prepared by adding different types of nanoparticles exhibit superior thermophysical properties and functional characteristics. In this paper, we prepared a water-based magnetic fluid loaded with multi-walled carbon nanotubes (MCNTs), silver (Ag), and copper (Cu) to enhance thermal conductivity. Using a transient double hot-wire method, we designed and built an experimental measurement system for the thermal conductivity of magnetic fluids with an average measurement error of less than 5%. We studied the thermal conductivity of hybrid magnetic fluids under different conditions and evaluated the advantages and disadvantages of various models, including the Maxwell model, H&C model, Tim model, Y&C model, and Evans model. Our results show that MF+MCNTs, MF+Ag, and MF+Cu nanofluids can all improve the thermal conductivity of the carrier fluid, with MF+MCNTs exhibiting the best improvement effect of 10.93%. Among the five models evaluated, the Evans model had the best predictive effect with a deviation range within 5%. This work provides theoretical and practical reference for enhancing the thermal conductivity of magnetic fluids and provides a more accurate theoretical model for calculating the thermal conductivity of hybrid magnetic fluids.

## 1. Introduction

Magnetic fluid (MF), also known as ferrofluid, is a colloidal suspension usually formed by ferromagnetic nanoparticles uniformly suspended in a non-magnetic carrier fluid using a surfactant. It can exhibit flowability like any other conventional fluid and magnetic properties similar to those of other solid magnetic materials in the presence of a magnetic field. Therefore, magnetic fluid has been applied in the fields of sealing, energy transport, shock absorption, sensors, etc. In general, the rate of heat transfer through a material is influenced by its thermal conductivity, temperature gradient, and cross-sectional area. Materials with high thermal conductivity, such as nanofluids, are able to transfer heat more efficiently than those with low thermal conductivity. This makes them ideal for use in applications where intensive heat transfer is required [1,2]. The thermal conductivity of fluids is a critical parameter for many applications that require efficient heat transfer [3]. However, conventional fluids often have low thermal conductivity and are in need of improvement through the addition of other components.

There is considerable research showing that magnetic fluids have improved thermal conductivity. Zhu et al. [4] created Fe_3_O_4_ magnetic nanofluids using distilled water and analyzed the impact of volume fractions on thermal conductivity improvement. Yu et al. [5] reported a 34% increase in thermal conductivity for a Fe_3_O_4_ nanofluid in kerosene with a concentration of 1%. Abareshi et al. [6] conducted a study on the thermal conductivity of magnetic nanofluids as a function of their volume fraction and temperature and found that both factors contributed to an increase in the thermal conductivity ratio. The highest enhancement was 11.5% for a nanofluid with 3 vol% nanoparticles at a temperature of 40 °C. Syam Sundar et al. [7] conducted a study on the thermal conductivity and viscosity of a magnetic nanofluid composed of Fe_3_O_4_ and water and observed an increase in both properties associated with an increase in volume concentration. Philip et al. [8,9] investigated the thermal conductivity of magnetic nanofluids (MNFs) created using magnetite nanoparticles dispersed in carrier fluids such as water, ethylene glycol, and kerosene. They found that the ratio of thermal conductivity increased with particle volume fraction. However, no improvement was seen below 1.71 vol%. The linear phase had a gradient of 0.035 and the highest ratio observed was 23% at 7.8 vol%. Hong et al. [10] performed two studies on the thermal conductivity enhancement of ethylene glycol-based magnetic nanofluids. In one of their studies, they observed that the thermal conductivity ratio increased in a non-linear manner with volume fraction. In another investigation [11], they compared the thermal conductivity of nanofluids with different iron nanoparticle volume fractions in ethylene glycol and found that it increased with particle volume fraction. Comparing copper and iron nanoparticles dispersed in ethylene glycol, they found that the thermal conductivity enhancement was greater in iron-based nanofluids than in copper-based nanofluids. Bahiraei et al. [12] investigated the thermal conductivity and viscosity of water–Fe_3_O_4_ nanofluid in an experimental study at volume concentrations of 0–4% and temperatures of 25–60 °C. They designed and constructed a test apparatus for determining the thermal conductivity. Their findings indicated that both thermal conductivity and viscosity increased with increased volume concentration. Additionally, an elevation in temperature resulted in a decline in viscosity and a rise in thermal conductivity. It was discovered that the link between thermal conductivity and viscosity concerning concentration was non-linear.

A large number of investigations have been carried out on nanofluids containing single-type nanoparticles, and the properties of these colloidal mixtures have been extensively characterized and studied [13,14]. More recently, there has been an increase in interest in the evaluation of new hybrid ferrofluids, which are combinations of different nano-additives, in order to improve heat transfer rates. Advances in nanocomposites have enabled hybrid nanomaterials (nanoparticles) to be fabricated, and leading researchers are currently investigating the properties of hybrid nanofluids. Hybrid nanofluids have a wide range of applications in the fields of thermal engineering, energy, biomedicine, electronics, environment, etc., due to their excellent thermophysical properties and functional characteristics that single nanofluids do not possess. Hybrid nanofluids can be made via two main methods: (a) the addition of two different types of nanoparticles to a base fluid or (b) the preparation of nanocomposites and their subsequent dispersion in a base fluid. Nanocomposites have been used by the majority of researchers for the synthesis of hybrid nanofluids. However, researchers generally obtain hybrid nanofluids by loading two different nanoparticles into a base fluid and subjecting them to ultrasonic dispersion, due to the high cost of using nanosynthetic materials. Carbon nanotubes, copper, silver, etc., are often chosen as nanocomposite materials because of their high thermal conductivity.

Jha and Ramaprabhu [15] carried out measurements of the thermal conductivity of nanofluids based on multi-walled carbon nanotubes (MWCNTs) and Cu-decorated MWCNTs. The authors reported that higher thermal conductivity was observed at lower MWCNT loadings in the base fluid, which may be due to more homogeneous particle dispersion under these conditions. The study also indicated that the thermal conductivity enhancement achieved with Cu-decorated MWCNT-based nanofluids was more significant than that reported by Eastman et al. [16] For example, increases of 35.2% and 10.1% in thermal conductivity were observed for distilled water and ethylene glycol, respectively, when only 0.03 vol% of nanocomposites were added. Sundar et al. [17] reported that the enhancement of thermal conductivity in nanofluids is due to the micro-convection and Brownian motion of the particles in the base fluid. They observed a 13.88% increase in thermal conductivity for 0.3% MWCNT–Fe_3_O_4_ based nanofluids in comparison to the base fluid at a temperature of 20 °C. At a higher operating temperature of 60 °C, the increase in thermal conductivity was even higher and reached 28.46%. Harandi et al. [18] presented an experimental study on temperature and concentration effects on the thermal conductivity of f-MWCNT–Fe_3_O_4_/EG hybrid nanofluids. The experiments were carried out at temperatures between 25 °C and 50 °C for solid volume fractions ranging from 0 to 2.3%. Their results showed that the ratio of the thermal conductivity increased with increases in the solid volume fraction and the temperature. They also found that the variation in the thermal conductivity ratio with the solid volume fraction was greater at higher temperatures than it was at lower temperatures. In addition, at higher solid volume fractions, the effect of temperature on the thermal conductivity ratio was more pronounced. Lee et al. [19] made hybrid water-based nanofluids containing TMAH-coated Fe_3_O_4_ nanoparticles and GA-coated CNTs and then measured their thermal conductivity and viscosity. Their results showed that with an increasing concentration of either Fe_3_O_4_ nanoparticles or CNTs, both viscosity and thermal conductivity increased. In addition, they found that the thermal conductivity increased with increasing temperature while the viscosity decreased. Sundar et al. [20] prepared stable magnetic nanofluids by dispersing nanodiamond–Fe_3_O_4_ nanocomposites in water and ethylene glycol/water mixtures and experimentally measured the effects of particle loading and temperature on the thermal conductivity and viscosity. Their results showed that, for water, as well as for 20%:80%, 40%:60%, and 60%:40% EG/W-based nanofluids at 0.2% volume concentration at a temperature of 60 °C, the thermal conductivity improvements were 17.8%, 13.4%, 13.6%, and 14.6%, respectively. Du et al. [21] studied Fe_3_O_4_ and multi-walled carbon nanotubes (MWCNTs) mixed and dispersed in water-based fluids at temperatures ranging from 25 °C to 50 °C and volume fractions ranging from 0.2% to 1.0%. Thermal conductivity enhancements (TCE) of 32.76% and 33.23% were measured for the mono-nanofluid (MN) and the hybrid nanofluid (HN), respectively, at a temperature of 50 °C and a volume fraction of 1.0%. Batmunkh et al. [22] attempted to improve the thermal conductivity of TiO_2_ nanofluids by combining flat “Ag” particles with small (15 nm) and large (300 nm) TiO_2_ nanoparticles in aqueous solutions. Over a temperature range of 15 to 40 °C, the thermal conductivity of Ag/TiO_2_ water nanofluids with different weight concentrations was measured. Their study shows that the thermal conductivity of TiO_2_-based solutions can be improved by the introduction of flat “Ag” particles. Ebrahimi et al. [23] carried out an experimental study of the thermal conductivity of water-based Fe_3_O_4_/CuO nanofluids as a function of various parameters. Their results indicate that the thermal conductivity of Fe_3_O_4_ nanofluids is significantly increased by the presence of different concentrations of CuO nanoparticles. A linear relationship between the concentration of CuO nanoparticles and the thermal conductivity of Fe_3_O_4_ nanofluids was observed over a temperature range of 25 to 50 °C.

In summary, previous research has investigated the thermal conductivity of nanofluids containing single types of nanoparticles as well as hybrid nanofluids. Mono-nanofluids have been extensively studied to improve their thermal conductivity by using different types of nanoparticles under different conditions. In contrast, studies on hybrid nanofluids have mainly focused on the production of nanofluids during preparation by the combination of two types of nanoparticles. However, there have been few studies on the loading of different high-thermal-conductivity nanoparticles onto nanofluids that can be stably dispersed. While some studies have reported improved thermal conductivity in polydisperse nanofluids compared to their monodisperse counterparts, others have witnessed a decline in thermal conductivity caused by compatibility issues between different types of nanoparticles. Therefore, the thermal conductivity of hybrid magnetic fluids loaded with various nanoparticles remains uncertain and requires specific analyses for each nanofluid composition and condition. In this study, we formulated water-based magnetic fluids containing carbon nanotubes and silver and copper nanoparticles. By varying the temperature and mass fraction of magnetic fluids, this study investigated the impact of different nanoparticles on the thermal conductivity of water-based magnetic fluids. The resulting thermal conductivity data were compared with several theoretical models and their corresponding equations. Based on the experimental data, a generalized correlation was proposed for the studied ranges.

## 2. Materials and Methods

### 2.1. Materials

**Figure 1:**
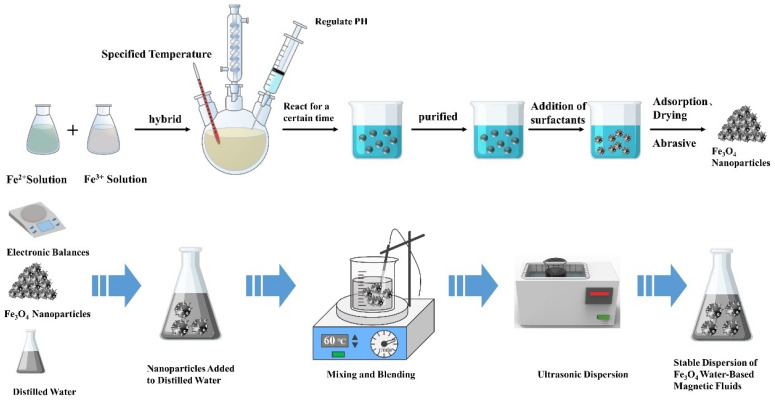
Water-based magnetic fluid preparation process.

**Figure 2:**
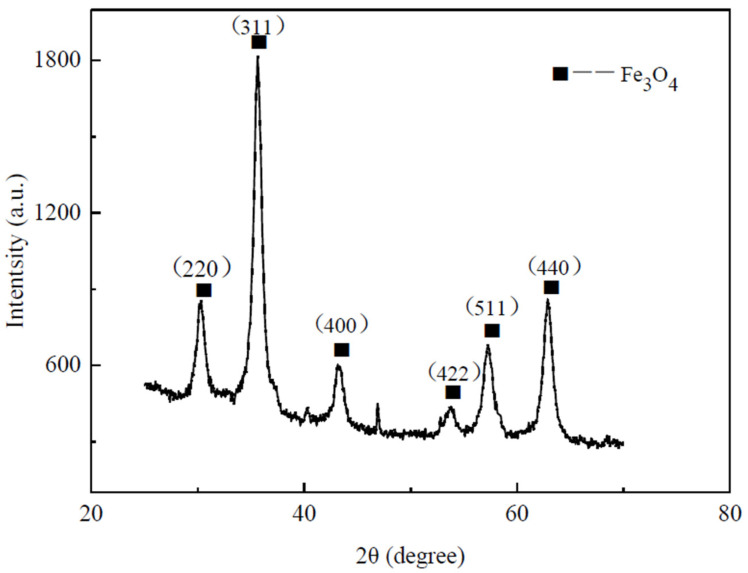
X-ray diffraction of synthesized Fe_3_O_4_ particles.

The water–Fe_3_O_4_ magnetic fluid investigated in this study was synthesized via chemical coprecipitation by our group. The process of preparing water-based magnetic fluid is presented in Figure 1. A precise amount of FeCl_2_–4H_2_O and FeCl_3_–6H_2_O were weighed in a 1:2 ratio, mixed together, and dissolved in deionized water. The solution was then transferred to a three-necked flask, which was heated to 70 °C using a thermostatic water bath and stirred using an electric stirrer at 300 rpm. The solution was kept constantly stirred at 300 rpm and heated at 70 °C using a thermostatic water bath. We then added an appropriate amount of NH_3_–H_2_O or NaOH solution to the salt mixture under continuous stirring for 30 min. This resulted in the creation of a black precipitate. Oleic acid was added to the solution as a surfactant and the reaction was allowed to proceed for an hour. Upon completion, the heating and stirring were halted, and the resulting black mixture was transferred to a beaker. The beaker was then cooled and washed repeatedly with deionized water. The solid particles were washed, settled in a magnetic field, and subsequently dried in a vacuum oven. Finally, the particles were ground to produce Fe_3_O_4_ magnetic particles coated with a surfactant. The Fe_3_O_4_ magnetic nanoparticles coated with surfactant were homogeneously blended with the carrier liquid. The magnetic particles were evenly dissolved in the carrier liquid through ultrasonication and dispersion (or high-energy ball milling). The use of ultrasonic dispersion (or high-energy ball milling) ensured even dissolution of magnetic particles in the carrier liquid, thus generating a stable magnetic fluid. The Fe_3_O_4_ magnetic particles were analyzed using a Bruker D8-Advance X-ray diffractometer (XRD), and their XRD pattern is presented in Figure 2. Comparison with the standard PDF card revealed that Fe_3_O_4_ was the principal constituent of the product.

**Figure 3:**
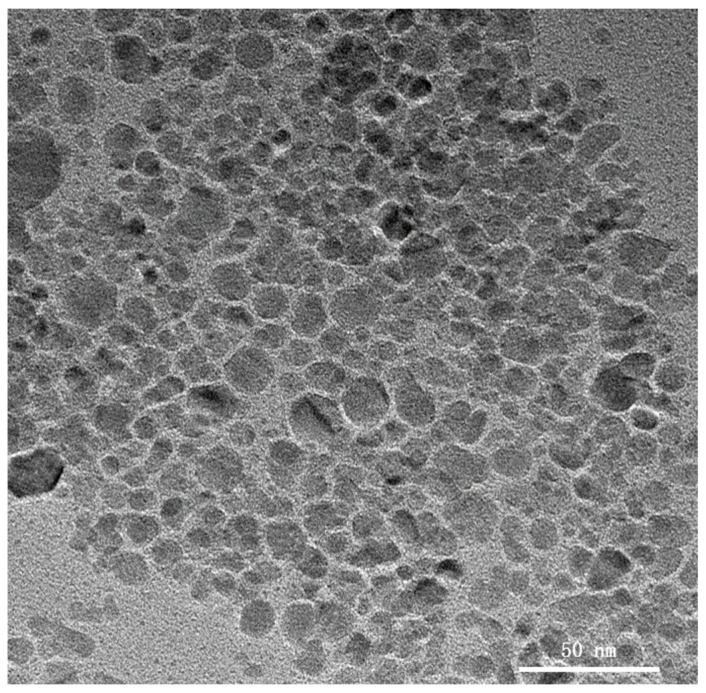
TEM image of Fe_3_O_4_ nanoparticles.

The water–Fe_3_O_4_ magnetic fluid sample exhibited stable performance for several years without visible particle agglomeration. And it is commercially available in magnetic fluid seals [24]. Transmission electron microscopy (TEM) images obtained using a JEM-2100F instrument revealed uniformly dispersed, nearly spherical particles with an average size of approximately 10 nm (Figure 3). Particles of this size range can be stably suspended in carrier fluids without sedimentation. Selected physical properties of the ferrofluid are presented in Table 1.

**Figure 4:**
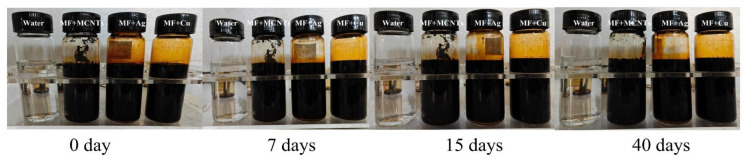
Observations on the stability of 2 wt% hybrid magnetic fluids.

The prepared magnetic fluid was loaded with different types of nanoparticles, such as MCNTs and Cu and Ag nanoparticles. Pristine multi-walled carbon nanotubes (MCNTs) and copper (Cu) and silver (Ag) nanoparticles produced by chemical vapor deposition were purchased from Beijing Zhongkeleiming Daojin Technology Co., Ltd. (Beijing, China). The primary traits of the nanoparticles are illustrated in Table 2, furnished by the suppliers of said nanoparticles. As shown in Figure 4, the hybrid magnetic fluids after being subjected to ultrasonic dispersion showed good stability. The weight fraction in this article denotes the proportion by weight of the nanoparticles in relation to the total weight of both the magnetic fluid and the nanoparticles. More details of the finished samples are shown in Table 3.

### 2.2. Thermal Conductivity Measurement Method

**Figure 5:**
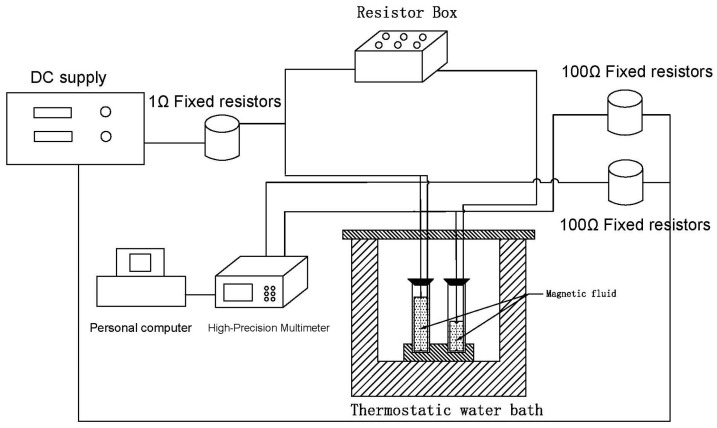
Experimental measurement system.

The thermal conductivity of the water–Fe_3_O_4_ magnetic fluid was measured using a transient hot-wire technique. An experimental setup, depicted schematically in Figure 5, was developed for this purpose. The setup consisted of a hot wire, a magnetic fluid container, a power source, a Wheatstone’s bridge, a data acquisition system, and a constant-temperature bath. A platinum wire with a length of 0.65 cm and a diameter of 0.2 mm, insulated with Teflon, served as the hot wire.

A water bath was used to investigate the effect of temperature on the thermal conductivity of the magnetic fluids. The temperature of the water in the bath was monitored at regular intervals and automatically adjusted by the controlling system to restrict deviations in temperature to less than 0.1 °C. A temperature sensor was placed on one side of the glass container to ensure that the temperature of the magnetic fluid inside remained constant by timely controlling the temperature of the water bath.

In the transient hot-wire method, the thermal conductivity of a fluid is determined by measuring the rate of temperature increase of a hot wire over time following the application of a step change in voltage. The entire apparatus is isolated from all sources of vibration to ensure that the test fluid, suspended with microfilaments, remains in a static state. The experimental measurement formula is presented in Equation (1):

(1) $$\lambda =\frac{{\left(I/2\right)}^{3}R\left(T\right)R\left(0\right)\alpha }{4\pi L}/\frac{dU}{d\ln t}$$

where I denotes the constant current supplied by the DC power source, *R*(0) and *R*(*T*) represent the resistance of the platinum wire at 273.15 K and temperature T, respectively, $\alpha =0.003761\;{{}^{\circ}C}^{-1}$ is the temperature coefficient of resistance for the platinum wire, determined through experimental calibration, *L* is the difference in length between the two hot wires, $d\left(U\right)$ is the bias voltage across the Wheatstone bridge, and t represents time.

**Figure 6:**
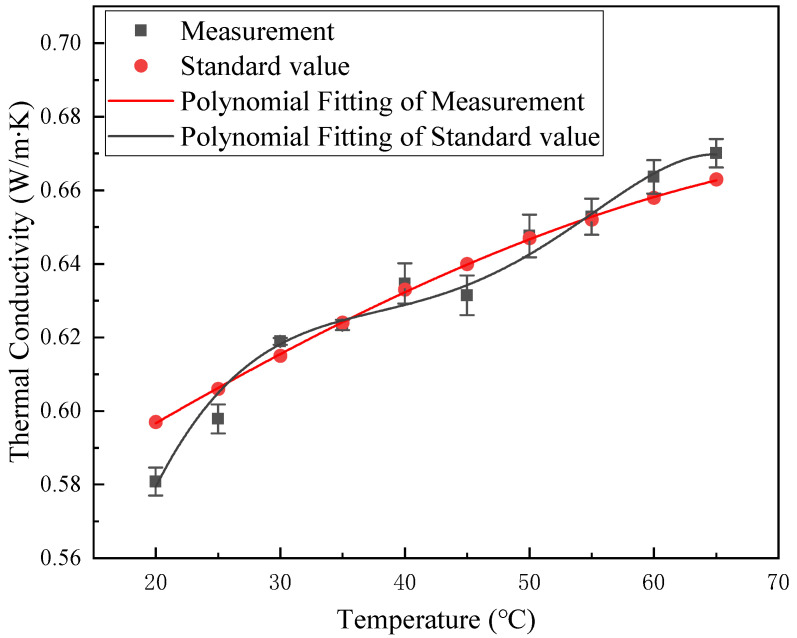
Validation of measurement device results with standard results [25].

To ensure the validity of the setup’s performance, the thermal conductivity of pure water was measured at different temperatures and compared with reliable data [25]. As shown in Figure 6, the measured values demonstrate good agreement with standard values, deviating by no more than 5%. A polynomial fit was performed on the measurements and the goodness of fit was *R*^2^ = 0.98. Similarly, a polynomial fit was made to the standard value of distilled water and the goodness of fit *R*^2^ = 0.99. The standard deviation of the results of the three repetitions of the test was calculated and solved to obtain the error bars, which are plotted in Figure 6.

### 2.3. Theoretical Thermal Conductivity Model of Magnetic Fluid

Nanofluid thermal conductivity models primarily use classical models, with Brownian models having widespread use in the field of nanofluid applications. Though there are numerous models available, adequacy and appropriateness are still topics of controversy due to conflicting thermal conductivity data. The disparity should be attributed to particle size, shape, temperature, volume concentration, and the static and dynamic conditions of nanoparticles.

The Maxwell model [26], which is a conventional model used to forecast thermal conductivity in mixtures containing a solid and a liquid, offers an equation for the efficient thermal conductivity ($\lambda_{\mathrm{nf}}$) for such systems:

(2) $$\lambda_{\mathrm{nf}}=\frac{\lambda_{\mathrm{np}}+2\lambda_{\mathrm{bf}}+2\left(\lambda_{\mathrm{np}}-\lambda_{\mathrm{bf}}\right)\phi }{\lambda_{\mathrm{np}}+2\lambda_{\mathrm{bf}}-\left(\lambda_{\mathrm{np}}-\lambda_{\mathrm{bf}}\right)\phi }\lambda_{\mathrm{bf}}$$

where $\lambda_{\mathrm{np}}$ is the thermal conductivity of the nanoparticle, $\lambda_{\mathrm{bf}}$ is the thermal conductivity of the base fluid, and *ϕ* is the particle volume fraction of the suspension. According to Maxwell’s formula, the effective thermal conductivity of nanofluids is dependent on the thermal conductivity of both the nanoparticles and the base fluid as well as the volume fraction of solid particles present.

Another model worth mentioning is the Hamilton–Crosser model [27], an improved version of the Maxwell model, which takes into account the shape of the particles, and the equation is presented below:

(3) $$\lambda_{\mathrm{nf}}=\lambda_{\mathrm{bf}}\left[\frac{\lambda_{\mathrm{np}}+\left(n-1\right)\lambda_{\mathrm{bf}}-\left(n-1\right)\phi \left(\lambda_{\mathrm{bf}}-\lambda_{\mathrm{np}}\right)}{\lambda_{\mathrm{np}}+\left(n-1\right)\lambda_{\mathrm{bf}}+\phi \left(\lambda_{\mathrm{bf}}-\lambda_{\mathrm{np}}\right)}\right]$$

where *n* is the form factor (for sphere, *n* = 3; for cylinder, *n* = 6)

Yu and Choi [28] modified the classical Maxwell and Hamilton–Crosser models to take into account the effect of a solid-like layer, which has a higher thermal conductivity than the surrounding liquid, on the surface of the nanoparticles.

(4) $$\lambda_{\mathrm{nf}}=\frac{\lambda_{\mathrm{npe}}+2\lambda_{\mathrm{bf}}+2\left(\lambda_{\mathrm{npe}}-\lambda_{\mathrm{bf}}\right){\left(1+\beta \right)}^{3}\phi }{\lambda_{\mathrm{npe}}+2\lambda_{\mathrm{bf}}-\left(\lambda_{\mathrm{npe}}-\lambda_{\mathrm{bf}}\right){\left(1+\beta \right)}^{3}\phi }\lambda_{\mathrm{bf}}$$

(5) $$\lambda_{\mathrm{nep}}=\frac{\left[2\left(1-\gamma \right)+{\left(1+\beta \right)}^{3}\left(1+2\gamma \right)\right]\gamma }{-\left(1-\gamma \right)+{\left(1+\beta \right)}^{3}\left(1+2\gamma \right)}\lambda_{\mathrm{np}}$$

where *γ* represents the ratio of the thermal conductivity of the nanolayer to the thermal conductivity of the particle layer, and *β* represents the ratio of the thickness of the nanolayer to the radius of the original particle.

In addition to classical models, Timofeeva et al. [29] proposed a mathematical model for calculating the thermal conductivity of nanofluids, based on a simple correlation derived from effective medium theory:

(6) $$\lambda_{\mathrm{nf}}=\left(1+3\phi \right)\lambda_{\mathrm{bf}}$$

Evans et al. [30] conducted molecular dynamics simulations to examine nanofluids’ thermal conductivity. Their resulting model is presented below:

(7) $$\lambda_{\mathrm{nf}}=\lambda_{\mathrm{bf}}\left(1+3\phi \frac{\varepsilon -1}{\varepsilon +2}\right)$$

where *ε* = r_p_/h is the ratio of the particle radius r_p_ to the equivalent matrix thickness h. For the purpose of analysis, a constant value of 2.9 was assumed for this ratio, representing the median of the boundaries utilized in the development of the model. Furthermore, since the particle radii and composition were constant in each independent dataset analyzed, the ratio *ε* was also maintained as a constant value within those datasets.

## 3. Results and Discussion

The objective of this study is to explore the relative thermal conductivity of hybrid magnetic fluids using the results of the experimental section. The impact of the three independent variables—namely temperature, the type of nanoparticles, and their weight fractions—was also investigated, along with the predictive accuracy of the classical model. The results are divided into four subsections, outlined below.

### 3.1. Influence of Temperature on the Thermal Conductivity

To better elucidate the mechanism underlying the enhancement of thermal conductivity, we define thermal conductivity ratio in Equation (8):

(8) $$\mathrm{TCR}=\frac{\Delta \lambda }{\lambda }=\frac{\lambda_{\mathrm{HYMF}}}{\lambda_{\mathrm{MF}}}\times 100\%$$

where TCR is thermal conductivity ratio, $\lambda_{\mathrm{HYMF}}$ is the thermal conductivity for hybrid magnetic fluids, and $\lambda_{\mathrm{MF}}$ is that of magnetic fluids.

**Figure 7:**
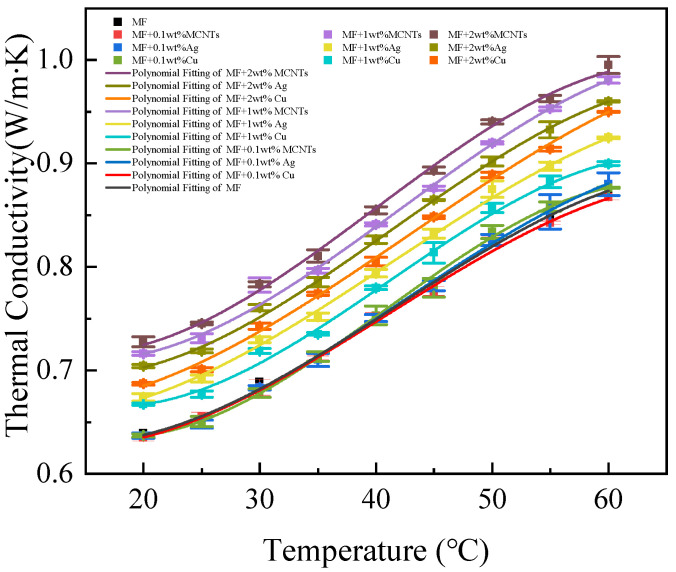
Variation in thermal conductivity with temperature for different nanoparticle types and their respective concentrations implemented in hybrid magnetic fluids (The error bars indicate the standard deviation of the three experiments).

Figure 7 illustrates the relationship between thermal conductivity and temperature for magnetic fluids containing different weight fractions and types of nanoparticles. A polynomial was fitted to the measurements for each sample with a goodness of fit of *R*^2^ = 0.99. And the three experimental measurements were calculated to obtain the standard deviation, which is plotted as an error bar in Figure 7. As temperature increases from 20 °C to 60 °C, thermal conductivity exhibits an approximately linear increase, consistent with trends observed in magnetic fluids. The maximum thermal conductivity is observed at 60 °C, while the minimum value occurs at 20 °C. At a nanoparticle weight fraction of 0.1%, thermal conductivity increases by an average of 37%; at a weight fraction of 1%, the increase is 36%; and at a weight fraction of 2%, the increase is 36%. The improvement in relative thermal conductivity following a temperature increase can be attributed to Brownian motion, according to the principle of kinetic theory [31]. This enhancement in thermal conductivity is attributed to the value of $\phi C_{N}v_{N}l/3$, where $C_{N}$ represents the heat capacity per unit volume of nanoparticles, $V_{N}$ denotes the root-mean-square velocity of the Brownian particle, and l is the mean free path. As nanoparticle aggregation occurs, resulting in the formation of larger aggregates or chains, there is a substantial reduction in convection velocity ($V_{N}$) due to its cubic dependence on particle size and linear dependence on the square root of temperature (T). As a result, an analogous increase in thermal conductivity is also anticipated, independent of the fluid used.

**Figure 8:**
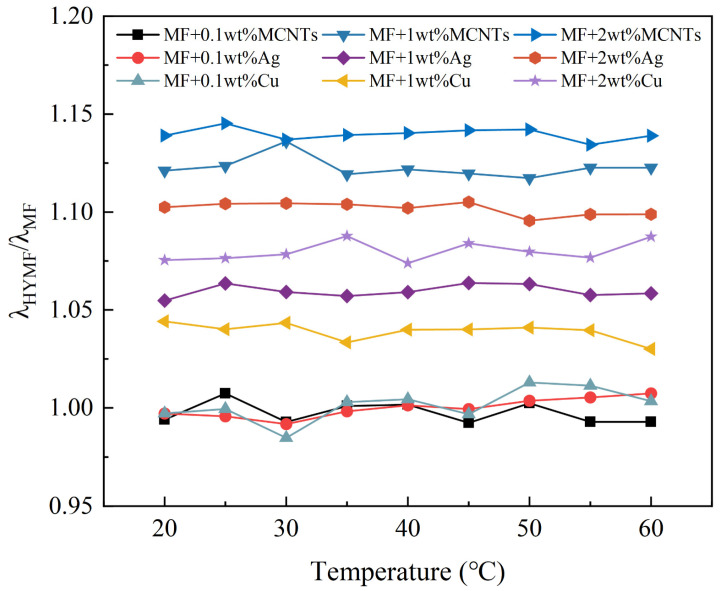
The variation in TCR with temperature for hybrid magnetic fluids.

Figure 8 depicts the relationship between the thermal conductivity ratio and temperature for magnetic fluids containing different weight fractions and types of nanoparticles. The outcomes show that the TCR remains constant as the temperature rises from 20 °C to 60 °C. These results suggest that the thermal conductivity (λ) of nanofluids closely tracks that of the base fluid. This trend of rising thermal conductivity with temperature has been detected in magnetic fluids based on both water and oil [3,32].

According to micro-convection models, the time necessary for a Brownian particle to travel its own diameter ($t_{B}$) is determined by the equation $3\pi \eta d^{3}/2k_{B}T$, where *η* denotes the viscosity of the base fluid, d represents the diameter of the nanoparticle, T is the absolute temperature, and $k_{B}$ is the Boltzmann constant. Research on dye diffusion showcases that although Brownian movement does not boost mass transport directly, it increases fluidic nanoscale stirring, thus augmenting convection currents. Brownian models assume a direct correlation between the thermal conductivity of nanofluids and the self-diffusion coefficient of nanoparticles. If micro-convection were found to enhance thermal conductivity, a temperature-dependent increase in the $\lambda_{\mathrm{HYMF}}/\lambda_{\mathrm{MF}}$ ratio would be anticipated. However, our experiments demonstrate a constant $\lambda_{\mathrm{HYMF}}/\lambda_{\mathrm{MF}}$ ratio, suggesting a less significant role for micro-convection in improving thermal conductivity.

### 3.2. Influence of Weight Fractions on the Thermal Conductivity

**Figure 9:**
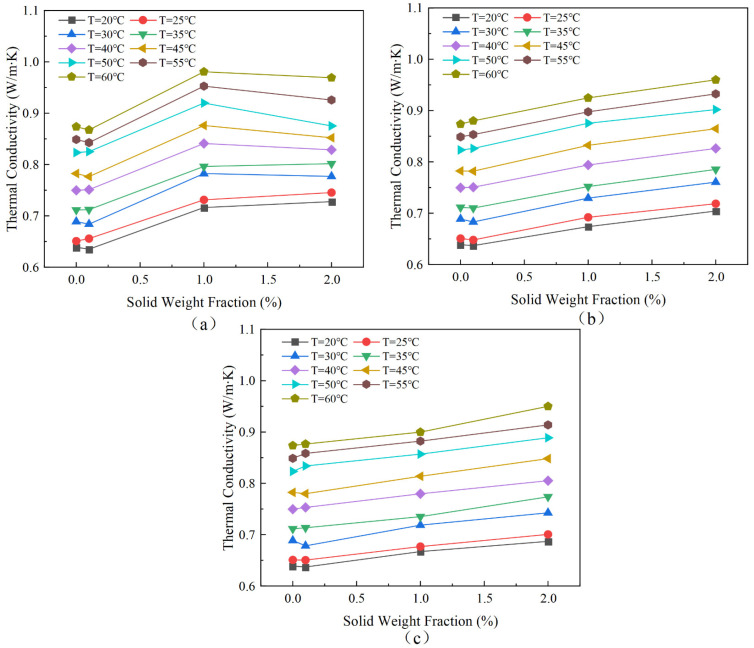
Variation in thermal conductivity with nanoparticle concentrations for hybrid magnetic fluids with different nanoparticle types and at different temperatures. (**a**) MF+MCNTs; (**b**) MF+Ag; (**c**) MF+Cu.

Figure 9 shows the thermal conductivity of hybrid magnetic fluids for various nanoparticle concentrations at a fixed Fe_3_O_4_ concentration. The experimental data demonstrate a monotonic increase in enhancement ratios for suspensions as a function of nanoparticle volume fraction. For loaded MCNTs, at a temperature of 60 °C, the thermal conductivity of MF + 0.1 wt% MCNTs increases by a maximum of 11%. For loaded Ag nanoparticles, the thermal conductivity of MF + 0.2 wt% Ag increases by a maximum of 10% at 60 °C. Similarly, for loaded Cu nanoparticles, the thermal conductivity of MF + 0.2 wt% Cu increases by up to 9% at 60 °C.

At higher weight fractions, the number of suspended nanoparticles increases, which could result in a rise in surface-to-volume ratio and particle collisions. When there is a significant number of particles, the influence of temperature on particle motion becomes more evident. Shahsavar et al. [33] similarly noted an increased thermal conductivity of Fe_3_O_4_/CNT hybrid magnetic fluids with the incorporation of nanoparticles.

At low concentrations, the rise in thermal conductivity is negligible as a result of the assembly of nanoparticles into alignments and small clusters. As depicted in the illustration below, the inclusion of MCNTs substantially enhanced the thermal conductivity when the hybrid magnetic fluid concentration ranged from 0–1%. As the concentration of nanoparticles increased, the quantity and length of these alignments grew, leading to an upsurge in thermal conductivity. This occurrence can be ascribed to the clustering of nanoparticles, which have high surface energies due to their large surface-to-volume ratio. The creation of chain-like networks of small nanoparticles enhances the volume fraction of effective heat-conducting phases in magnetic fluids [34]. These findings support the simulation results reported by Zhu [4]. These results demonstrate that both parallel and random alignments can increase the thermal conductivity of magnetic fluids by creating an extended path for heat transfer. The recorded alignments, not previously reported in other magnetic fluids, are thought to explain the notably increased thermal conductivity improvement in Fe_3_O_4_ magnetic fluids.

However, the peculiar tubular structure of MCNTs causes the formation of small, dispersed clusters at lower concentrations. As the concentration of nanoparticles increases, the number and size of these formations also increase, leading to a considerable enhancement in thermal conductivity. Additionally, the concentration is within the range of 1% to 2%. When the concentration of nanoparticles increases, it results in the formation of significant and condensed clusters in the liquid. As a result, a substantial amount of particle-free liquid is produced, which exhibits a high degree of thermal resistance. Consequently, it offsets the enhancement caused by the increase in the nanoparticle concentration, deviating the thermal conductivity from the linear increasing trend. In summary, a certain level of clustering may enhance thermal conductivity and excessive clustering creates an opposite effect due to associated sedimentation.

### 3.3. Influence of Nanoparticle Type on the Thermal Conductivity

**Figure 10:**
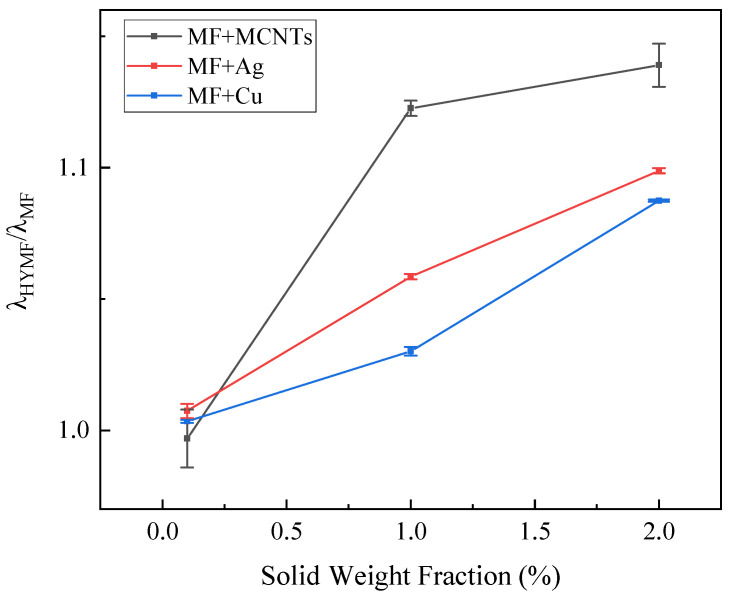
The variation in the thermal conductivity ratio with the mass fraction associated with loading different nanoparticles, at 60 °C.

As demonstrated in Figure 7, Figure 8, Figure 9 and Figure 10, the thermal conductivity of the hybrid magnetic fluids significantly increased at concentrations of 1 wt% and 2 wt% when compared to the base fluid. This enhancement can be credited to the notable thermal conductivity of MCNTs and Ag and Cu nanoparticles. These findings suggest that the hybrid magnetic fluids display better thermal conductivity than their single magnetic counterpart. And this aligns with the findings of Sundaret et al. [20]. Figure 10 illustrates an improvement in measured thermal conductivity, suggesting that loading high-thermal-conductivity nanoparticles boosts the thermal conductivity of magnetic fluids, maintaining a constant concentration. Out of the three hybrid magnetic fluids (MF+MCNTs, MF+Ag, and MF+Cu), MF+MCNTs shows the highest thermal conductivity under the same conditions. At a mass fraction of 2 wt%, the thermal conductivity of MF + 2 wt% MCNTs is improved by up to 12%. The corresponding enhancements for MF + 2 wt% Ag and MF + 2 wt% Cu are 10% and 9%, respectively. However, MF + 0.1 wt% MCNTs has a lower thermal conductivity value compared to the base fluid and this difference is due to measurement error. A concentration of 0.1% MCNTs should closely correspond to the thermal conductivity of the base fluid, so the thermal conductivity ratio should be close to 1. With the addition of the error bars to the graphs, the experimental results can be more accurately reflected.

Two primary factors underlie these experimental results. Firstly, the three different types of nanoparticles have distinct thermal conductivities, characterized by the order MCNTs > Ag > Cu. Adding high-thermal-conductivity nanoparticles to magnetic fluids leads to a noteworthy enhancement in their thermal conductivity. This demonstrates that materials with high thermal conductivity can improve the efficiency of heat transfer in fluids. Secondly, it was shown by Gu et al. [35] that the use of materials with a high aspect ratio can significantly increase the thermal conductivity of nanofluids. Our findings also support the idea that the cylindrical shape of multi-walled carbon nanotubes contributes to improved thermal conductivity. Additionally, our results indicate that cylindrical nanoparticles offer greater enhancements in thermal conductivity when compared to spherical particles. The primary difference between the two suspension systems relies on particle morphology, specifically size and shape. The particles’ shape affects the transfer of heat between the solid particles and the fluid base. Subsequently, it can be inferred that cylindrical nanoparticles enhance thermal conductivity more than spherical nanoparticles due to a larger surface area and superior heat transfer over longer distances, typically micrometers. These results align with Kim et al.’s research [36].

### 3.4. Comparative Analysis of Classical Models

In order to verify the accuracy of the classical theoretical model, we computed the deviation in the thermal conductivity ratio using Equation (9):

(9) $$\mathrm{MOD}=\frac{\lambda_{\mathrm{nf}}-\lambda_{f}}{\lambda_{f}}\times 100\%$$

where MOD is the margin of deviation, nf represents nanofluid, and f represents base fluid.

**Figure 11:**
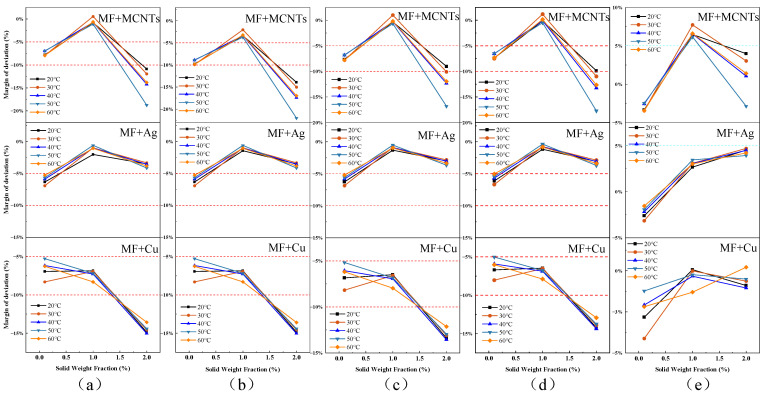
Comparison of deviation magnitudes of five models: (**a**) Maxwell model, (**b**) H&C model, (**c**) Timofeeva model, (**d**) Y&C model, (**e**) Evans model.

Assuming an acceptable deviation range of 10%, Figure 11a–c shows that the thermal conductivity values based on the EMT equation deviate within this range at mass fractions of 0.1% and 1%. However, the calculated values for MF + 2 wt% MCNTs and MF + 2 wt% Cu are significantly overestimated, particularly for MMCNTs due to their relatively high thermal conductivity, resulting in a maximum deviation of 17% with increasing concentration. In contrast, the theoretical calculation model accurately reflects the experimental values for MF + 0.1 wt% MCNTs and MF + 0.1 wt% Ag, with a minimum deviation of −0.2%. A comparison of calculated values for the three types of nanoparticles shows that the deviation range for MF+Ag remains within 10% at different concentrations and temperatures, accurately reflecting experimental values. One limitation of the EMT-based theoretical model is its inability to predict changes in thermal conductivity with temperature, with no significant changes observed for the three types of nanoparticles added at the same concentration for different temperatures. Figure 11d shows the deviation range between the Y&C theoretical model, which considers nanolayer structures, and experimental values, with a similar overall change range to those of the first three EMT-based models. The Y&C model is a modification of the Maxwell model, but its predictive power is limited by the empirical estimation of nanolayer thickness and thermal conductivity. In this study, we assumed that nanolayer thermal conductivity is equal to particle thermal conductivity (i.e., *γ* = 1, reducing to *λ*_npe_ = *λ*_np_) and *β* = 0.1, which could account for the higher bias and should be carefully considered when using correlation.

Figure 11e displays the deviation range between the thermal conductivity calculation model of Evans, based on molecular dynamics simulation, and experimental values. The deviation range for calculated values of magnetic fluids loaded with different concentrations of nanoparticles is within 10%, with a maximum of 7%. Compared to the other four models, the Evans model exhibits the highest prediction accuracy, with the highest calculation accuracy for MF+Ag and MF+Cu models, within a 5% deviation range and a minimum of 0.01%, closely matching experimental values. However, the Evans model also cannot accurately reflect the effect of temperature on thermal conductivity, as Evans et al. [30] used a kinetic theory-based analysis to demonstrate that hydrodynamic effects associated with Brownian motion have less impact on nanofluids, while thermal conductivity is only minimally affected and Brownian motion is highly dependent on fluid temperature.

As illustrated in the figure below, both the EMT-based theoretical model and the molecular dynamics-based model for thermal conductivity calculation have limitations. Both models were created for a single nanoparticle and, therefore, display varying degrees of deviation for different concentrations of the mixed magnetic fluids analyzed in this study. They are not well suited to actual experimental outcomes, which include fluctuations of 1% in weight fraction.

In summary, a comparison of the five models reveals that the Maxwell, H&C, and Tim models, all derived from EMT, generally overestimate calculated results. The Y&C model, based on a thermal conductivity model proposed for nanolayer structures, shows little difference from EMT-based calculated results. The Evans model, based on a theoretical calculation model proposed by molecular dynamics, considers the mechanism of microstructure heat conduction changes and exhibits a deviation range within an acceptable 10%, with the most accurate prediction effect. As shown in Table 4, the results of this experiment allow us to summarize the nanoparticles’ fraction ranges to which each model applies. In the future, we will conduct in-depth research to develop a new model that can more accurately reflect the thermal conductivity of hybrid magnetic fluids.

## 4. Conclusions

In this paper, the change in thermal conductivity under different conditions is mainly investigated after the addition of MCNTs and Ag and Cu nanoparticles to a sufficiently stable magnetic fluid for a long time. The thermal conductivity measurement system, designed and built based on the transient double hot-wire method, exhibits an uncertainty within 5%, meeting measurement requirements. Experimental results are compared with several thermal conductivity prediction models. The conclusions about thermal conductivity are as follows:

(1) Research has demonstrated that the thermal conductivity of magnetic fluids can be enhanced by the incorporation of highly thermally conductive nanoparticles. Among the various nanoparticles tested, carbon nanotube–magnetic fluid (MF+MCNTs) exhibited the highest thermal conductivity, followed by silver–magnetic fluid (MF+Ag) and copper–magnetic fluid (MF+Cu), under identical experimental conditions;
(2) The thermal conductivity of magnetic fluids has been shown to increase with the mass fraction of nanoparticles. Upon the addition of 1 wt% and 2 wt% solid particles, the thermal conductivity of carbon nanotube–magnetic fluid (MF+MCNTs) increased by 11.16%, while that of silver–magnetic fluid (MF+Ag) and copper–magnetic fluid (MF+Cu) increased by 10% and 8%, respectively;
(3) The thermal conductivity of hybrid magnetic fluids has been approximated as a linear increase with temperature from 20 °C to 60 °C. The thermal conductivity of carbon nanotube–magnetic fluid (MF+MCNTs) increased by 12%, while that of silver–magnetic fluid (MF+Ag) and copper–magnetic fluid (MF+Cu) increased by 10% and 9%;
(4) A comparison of classical theoretical models reveals their limitations in predicting the thermal conductivity of hybrid magnetic fluids. Only within a certain range can several models accurately predict thermal conductivity. However, the Evans molecular dynamics-based model demonstrates strong predictive ability for the magnetic fluids prepared in this experiment under specific conditions.

In the future, we will focus on the thermal conductivity of magnetic fluids mixed with different nanoparticles under the action of a magnetic field and strive to improve the thermal conductivity prediction model, laying a theoretical and experimental foundation for enhancing the heat transfer of magnetic fluids and their applications in thermal engineering.

## Figures and Tables

**Table 1:** Parameters of the examined magnetic fluid.

Samples	Temperature (°C)	$\mathrm{Density}\;\rho_{ff}$ (g/cm^3^)	Saturation Magnetization *M*s (kA/m)	Volume Fraction of Nanoparticles φ	$\mathrm{Viscosity}\;\mathrm{at}\;\mathrm{Zero}\;\mathrm{Fields}\;\eta_{0}$ (mPa.s)
Water-based magnetic fluid	25	1.16	13.51	3.7%	1.13

**Table 2:** Parameters of the examined nanoparticles.

Nanoparticle Type	Pipe Diameter/Grain Size (nm)	Lengths (μm)	Purity	Bulk Density (g/cm^3^)
MCNTs	8–15	50	98%	0.27
Ag	20	\	99.9%	0.5
Cu	20	\	99.9%	0.2

**Table 3:** Details of Finished Samples.

Sample Name	Nanoparticle Type	Nanoparticle Weight Fraction (%)
MF + 0.1 wt% MCNTs	MCNTs	0.1
MF + 1 wt% MCNTs		1
MF + 2 wt% MCNTs		2
MF + 0.1 wt% Ag	Ag	0.1
MF + 1 wt% Ag		1
MF + 2 wt% Ag		2
MF + 0.1 wt% Cu	Cu	0.1
MF + 1 wt% Cu		1
MF + 2 wt% Cu		2

**Table 4:** Applicable fraction ranges of different theoretical models [26,27,28,29,30].

Theoretical Model	Sample Name	Applicable Fraction Range
Maxwell	MF+MCNTs	0.1–1 wt%
	MF+Ag	0.1–2 wt%
	MF+Cu	0.1–1 wt%
Hamilton–Crosser	MF+MCNTs	0.1–1 wt%
	MF+Ag	0.1–2 wt%
	MF+Cu	0.1–1 wt%
Yu and Choi	MF+MCNTs	0.1–1 wt%
	MF+Ag	0.1–2 wt%
	MF+Cu	0.1–1 wt%
Timofeeva	MF+MCNTs	0.1–1 wt%
	MF+Ag	0.1–2 wt%
	MF+Cu	0.1–1 wt%
Evans	MF+MCNTs	0.1–2 wt%
	MF+Ag	0.1–2 wt%
	MF+Cu	0.1–2 wt%

## Data Availability

The numerical data used to support the findings of this study are included within the article.
